# Assessment of Immunological Features in Muscle-Invasive Bladder Cancer Prognosis Using Ensemble Learning

**DOI:** 10.3390/cancers13071624

**Published:** 2021-04-01

**Authors:** Christos G. Gavriel, Neofytos Dimitriou, Nicolas Brieu, Ines P. Nearchou, Ognjen Arandjelović, Günter Schmidt, David J. Harrison, Peter D. Caie

**Affiliations:** 1 School of Medicine, University of St Andrews, St Andrews KY16 9TF, UK; i.p.nearchou@gmail.com (I.P.N.); djh20@st-andrews.ac.uk (D.J.H.); pdc5@st-andrews.ac.uk (P.D.C.); 2 School of Computer Science, University of St Andrews, St Andrews KY16 9SX, UK; nd26@st-andrews.ac.uk (N.D.); oa7@st-andrews.ac.uk (O.A.); 3 Definiens GmbH, 80636 Munich, Germany; nicolas.brieu@gmail.com (N.B.); gschmidt@definiens.com (G.S.); 4 NHS Lothian, University Hospitals Division, Edinburgh EH16 4SA, UK

**Keywords:** immuno-oncology, tumour microenvironment, tumour budding, PD-L1, macrophages, lymphocytes, prognosis, survival analysis, machine learning, digital pathology

## Abstract

**Simple Summary:**

Muscle-invasive bladder cancer (MIBC) accounts for the majority of bladder cancer mortality worldwide. Clinical assessment of MIBC mainly relies on the TNM staging system to provide guidance for both prognosis and therapy planning. Based on standardized quantification of tumour-immune features across whole slide images, and in conjunction with clinical information, we construct an ensemble machine learning model that correctly classifies 71.4% of the patients who succumb to MIBC, significantly higher than the 28.6% of TNM staging system. Post-hoc analysis of our model reveals clinically relevant, immunological features for MIBC prognosis, thereby further supporting their adoption into the clinic.

**Abstract:**

The clinical staging and prognosis of muscle-invasive bladder cancer (MIBC) routinely includes the assessment of patient tissue samples by a pathologist. Recent studies corroborate the importance of image analysis in identifying and quantifying immunological markers from tissue samples that can provide further insight into patient prognosis. In this paper, we apply multiplex immunofluorescence to MIBC tissue sections to capture whole-slide images and quantify potential prognostic markers related to lymphocytes, macrophages, tumour buds, and PD-L1. We propose a machine-learning-based approach for the prediction of 5 year prognosis with different combinations of image, clinical, and spatial features. An ensemble model comprising several functionally different models successfully stratifies MIBC patients into two risk groups with high statistical significance (*p* value < $1\times 10^{-5}$). Critical to improving MIBC survival rates, our method correctly classifies 71.4% of the patients who succumb to MIBC, which is significantly more than the 28.6% of the current clinical gold standard, the TNM staging system.

## 1. Introduction

Urothelial cancer of the bladder (bladder cancer) is one of the most prevalent cancers worldwide, with approximately 430,000 new diagnoses each year [1]. The high morbidity and mortality rates, as well as the high socioeconomic burden, make bladder cancer a debilitating and often fatal disease [2,3]. Even though the majority of bladder cancer patients are diagnosed with non-muscle-invasive bladder cancer (NMIBC), recurrence and progression of the disease may lead to muscle-invasive bladder cancer (MIBC) [4]. Approximately 25% of newly diagnosed patients have MIBC, and unlike NMIBC, these tumours are biologically aggressive with limited therapeutic options. Although radical cystectomy with bilateral pelvic lymph node dissection is the current gold-standard treatment for MIBC, more than 50% of MIBC patients die from metastatic disease within 5 years [5]. To decrease the mortality rates, patients with a high risk of disease-specific death need to be identified more precisely, thereby allowing for better patient management and new treatments to be tested in the high-risk group.

Due to the intra- and inter-tumoural heterogeneity of MIBC, which is evident from the phenotypic and molecular diversity of tumour cells, choosing the most effective treatment for each patient is very challenging [6,7]. Currently, clinical assessment of bladder uses the Tumour-Node-Metastasis (TNM) staging system [8], where T describes the depth of invasion into the bladder wall, and N and M the presence or lack of node and distant metastasis, respectively. MIBC ranges from tumours which invade the detrusor muscle (T2), to tumours which spread to nearby organs (T4) [1,9]. Although TNM plays a critical role in guiding treatment planning, it remains an anatomy-based classification tool with patients of the same tumour stage experiencing a high variability in disease outcome [10,11].

The tumour mass comprises a heterogeneous population of cancer cells, and, together with a diverse group of resident and infiltrating host cells, they make up the tumour-immune microenvironment [12,13]. With the emergence of immuno-oncology, many cancer researchers have investigated the importance of the intra-tumoural host immune response within what is often an immunosuppressive tumour microenvironment [14,15]. Analysing the location, density, functional state, and organisation of the immune cell populations within the tumour landscape, often termed as the immune contexture, has become a fundamental step in identifying immune system characteristics that may be beneficial to patients [16]. In particular, an increasing number of studies has shown the critical role of the immune contexture (not yet included in the TNM guidelines) in patient survivability, suggesting that it could be a valuable determinant of patient prognosis [15,17]. Motivated by multiple papers [18,19,20,21], we have investigated the prognostic role of tumour-infiltrating lymphocytes (TILs), tumour-associated macrophages (TAMs), tumour buds (TBs), and programmed cell death-ligand 1 (PD-L1) in MIBC patients.

Lymphocytes and macrophages are generally found either infiltrating into or surrounding the tumour mass, both the core and the invasive front. TILs can be divided into subpopulations by virtue of their specialised functions, surface cluster of differentiation (CD) molecules and, in certain circumstances, morphological features. Cytotoxic T-cells are the main effector cells in the anti-tumour T-cell response, with a large volume of studies showing that their presence in the tumour-immune microenvironment is strongly associated with prolonged survival in various types of cancer [22,23]. TAMs have also been identified as decisive factors in the orchestration of the tumour-immune microenvironment [24]. They can exhibit polarised phenotypes, with classically activated M1 and alternatively activated M2 subpopulations possessing anti-tumoural and pro-tumoural abilities, respectively [25]. In particular, during metastasis, M2 macrophages are recruited at distinct pre-metastatic niches, where they can promote tumour cell dissemination and disrupt the function of TILs [24].

Tumour budding is generally considered to be the first step of cancer metastasis, defined as the dissociation of isolated single cancer cells or discrete clusters of up to four cancer cells, predominantly from the invasive front of the tumour [26]. In the last decade, tumour budding has been widely investigated as a marker of aggressive tumour behaviour, due to its association with adverse clinicopathological characteristics and the epithelial–mesenchymal transition [27,28]. As a result, tumour budding has been added to TNM as a supplementary prognostic factor for colorectal cancer [29]. However, without reliable quantitative methods, tumour-budding quantification is challenging, due to poor inter-observer consistency [30,31].

Immune checkpoints are cell-surface receptors expressed by immune cells that modulate immune responses [32]. Complex interactions between the immune system and cancer, including the manipulation of immune checkpoints such as programmed cell death 1 (PD-1), enable tumour cells to evade immune surveillance. Specifically, PD-L1, which is secreted by tumour cells, binds to PD-1 expressed on the surface of TILs and suppresses their function to ensure the growth and development of tumour cells [33]. Although our understanding of the intricate and dynamic relationship between tumour cells and host cells is increasing, further characterization of the precise impact of tumour cells on their surroundings is needed.

In recent years, machine learning (ML) methodologies have been widely utilized for the construction of predictive models based on biological features [34,35,36]. Nevertheless, the number of papers which have adopted ML methodologies for survival analysis is limited [34,36,37,38]. In this paper, we employ an ML methodology to investigate the prognostic role of TILs, TAMs, TBs, PD-L1, and other clinicopathological factors in MIBC. The contribution of this paper is fourfold. To the best of our knowledge, this is the first study reporting the labelling of entire MIBC tissue sections with multiple fluorescence immune markers as well as the quantification of immunological features across both the tumour core and the invasive front of whole-slide immunofluorescence images. In addition, using image, spatial, and clinical features, our ML methodology improves the accuracy of the 5 year prognosis for MIBC patients by a large margin when compared to the current gold standard, TNM. Lastly, our findings reinforce the importance of the immune contexture in cancer prognosis, thereby further supporting its adoption in the clinic.

## 2. Materials and Methods

### 2.1. Patients and Tissue Samples

Tissue specimens from patients with MIBC who underwent radical cystectomy at Royal Infirmary and Western General Hospital in Edinburgh between the years 2006 and 2013 were collated into a cohort. Patients were excluded from this study either due to incomplete clinical records, extensive tissue section artefacts, or data censoring. The final study cohort was comprised of 78 patients. Archived formalin-fixed paraffin-embedded (FFPE) tissue blocks presenting the deepest invasion of cancer were selected for each patient based on both macroscopic and microscopic examination of haematoxylin and eosin (H&E)-stained slides by a pathologist and a research scientist. The corresponding unstained tissue sections were collected from the NHS Lothian NRS BioResource Research Tissue Bank, conforming to protocols approved under the ethical status granted by the East of Scotland Research Ethics Service (Ethical Approval Ref: 10/S1402/33) and with written informed consent from all the patients. All experiments were performed in accordance with the relevant guidelines and regulations. Clinicopathological data that included age, sex and TNM stage status, along with survival data, were retrieved from the available electronic medical records. Patients were followed-up for a total time of 113 months, with a median survival time of 23.6 months. In order to maintain the anonymity of the patient information, the samples were de-identified prior to conducting this study.

### 2.2. Multiplex Immunofluorescence and Whole Slide Imaging

**Figure 1:**
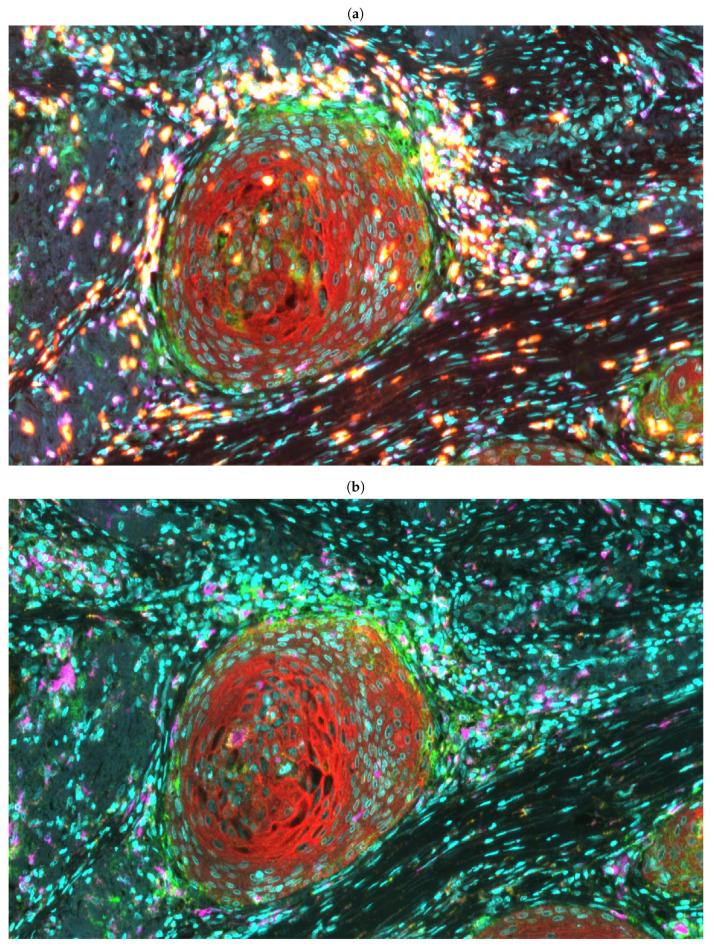
Examples of (**a**) TILs (Nuclei: Cyan, Cancer: Red, PD-L1: Green, CD3: Purple, CD8: Yellow) and (**b**) TAMs (Nuclei: Cyan, Cancer: Red, PD-L1: Green, CD68: Purple, CD163: Yellow) visualized using multiplexed immunofluorescence.

For each patient, automated tyramide-based immunofluorescence was performed on two de-paraffinised serial 3 μm thick sections of FFPE tissue mounted on superfrost plus slides using a Dako link 48 autostainer (Dako, Agilent Technologies). Primary antibodies against Pan-cytokeratin (PanCK), CD3, CD8, CD68, CD163 and PD-L1 were used to label urothelial cells, general T-cells, cytotoxic T-cells, M1/M2 (total) macrophages, M2 macrophages and immune checkpoint ligand PD-L1, respectively (see Supplementary Table S1 for further details about the primary antibodies). To increase the detection sensitivity and to visualise the target protein, tyramide signal amplification (TSA) reagents FITC, CY3 and CY5 were used for CD3 or CD68, PD-L1, and CD8 or CD163 respectively. Alexa Fluor 750 conjugated streptavidin antibody was used for the detection of PanCK. See Supplementary Table S2 for the detection and visualization reagents. Nuclei were counterstained with Hoechst (Hoechst 33342, Cat# H3570, ThermoFisher Scientific) and ProLong Gold Antifade mountant (Cat# P36930, ThermoFisher Scientific) applied directly to the tissue samples. The multiplex immuno-labeled tissue slides were scanned at 20× magnification and digitized into whole-slide fluorescence images using a Carl Zeiss AxioScan.Z1 scanner (Zeiss, Göttingen, Germany). Examples are shown in Figure 1. Supplementary Table S3 and Figure S1 show the excitation and emission wavelengths, along with the exposure time for each antibody used for multispectral imaging.

### 2.3. Detection of Cell Nuclei

For nucleus detection, the methodology described by Brieu and Schmidt [39] was adopted. For completeness, a high-level description of this methodology is provided. The methodology is comprised of four distinct stages: (a) A classification random forest (RF) was trained with long-range spatial context features [40] that were extracted from manually annotated foreground and background regions, where a foreground region contained the cell nucleus and vice-versa (n=4, resolution = 1200×1200 pixels). Subsequently, the RF generated a mask for all immunofluorescence (IF) images [41]. (b) A regression RF was trained to generate proximity maps using coordinates from manual annotations of cell nuclei (n=750 from 19 fields of view, resolution = 1485×1485). Intuitively, a proximity map enclosed the distance to the closest nucleus center for each pixel of the input image. (c) A regression RF was trained to generate surface area maps using manual annotations (n=250 from 8 fields of view, resolution = 580×580 pixels) [39]. A surface area model provides a mask of the initial image wherein each pixel is either zero, if it is not a part of a nucleus, or a positive real number, if it is part of a nucleus. The positive real number is the area of the corresponding nucleus. (d) At this stage, nuclei centers (see Supplementary Figure S2) are localized based on the proximity and surface area maps that were generated in (b) and (c) [39]. Note that both the original image and the corresponding mask (produced in (a)) are given as inputs to the models in (b) and (c), using only the PanCK and Hoechst IF channels. Data augmentation with varying scale, rotation, as well as intensity for both PanCK and Hoechst IF channels, was implemented in all stages. Finally, regions with necrotic tissue and any type of artefact, such as autofluorescence, were not included in any of the training data.

### 2.4. Segmentation of Epithelial Cells for the Identification of Tumour Buds

For the quantification of tumours buds, segmentation of the epithelial cells was required. The CNN-RF methodology described by Brieu et al. [42], and extended in [30], was adopted. Briefly, a convolutional neural network (CNN) was trained on an annotated dataset of epithelium and non-epithelium IF images. The dataset comprised 142×142 pixel regions, selected by an expert. The Hoechst, PanCK, CD3 and CD8 IF channels were normalised following the approach described by Brieu et al. [42]. An intensity interval was imposed on the PD-L1, CD163, and CD68 IF channels by computing the minimum and maximum values from the segmented epithelium regions of each slide. Once trained, the CNN produced a coarse segmentation mask of epithelium regions. Normalized PanCK, Hoechst, CD3, and CD8 channels of the IF images were used as input to the CNN. The predicted epithelium probability layer was used, together with the original IF channels, as input to a RF. Finer-grained segmentation masks were produced by the RF, enabling a more accurate segmentation of the epithelium. As detailed in previous work [30], the output of the CNN-RF is finally ensembled with the output of a semantic segmentation network [43] to generate the final epithelium segmentation results. TBs were classified as epithelium objects containing from one to four nuclei [30]. A total number of 97,262 patches were used for training and 9742 patches for validation of the CNN-RF model. The semantic segmentation network was trained with 19,093 patches and validated with 6987 patches of 256×256 pixels.

### 2.5. Cell Classification

For cell classification, given the normalised IF channels, a circular neighbourhood of each cell nuclei is defined (11×11 pixel radius) and the mean normalised intensity of the neighbourhood is computed for each IF marker (CD3, CD8, CD68, CD163, PD-L1 and PanCK). Cells are classified as positive or negative for a given IF marker if the corresponding mean normalized intensity is above or below a determined threshold, respectively. In our experiments, the threshold for all the IF markers was set to 32/256=0.125.

### 2.6. Pairwise Spatial Distributions of Lymphocytes, Macrophages, Tumour Buds and PD-L1

**Figure 2:**
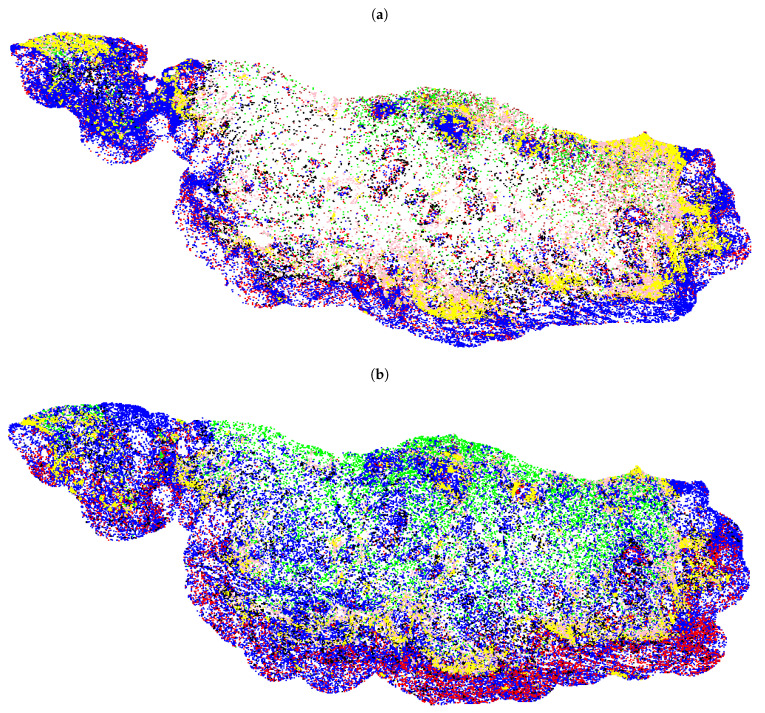
Nuclei localization of (**a**) TILs (CD3^+^PanCK^+^: Brown, CD3^+^PanCK^−^: Red, CD8^+^PanCK^+^: Green, CD8^+^PanCK^−^: Blue, TB: black, PD-L1^+^PanCK^+^: Pink, PD-L1^+^PanCK^−^: Yellow) and (**b**) TAMs (CD163^+^PanCK^+^: Brown, CD163^+^PanCK^−^: Red, CD68^+^PanCK^+^: Green, CD68^+^PanCK^−^: Blue, TB: Black, PD-L1^+^PanCK^+^: Pink, PD-L1^+^PanCK^−^: Yellow) across the WSI.

**Figure 3:**
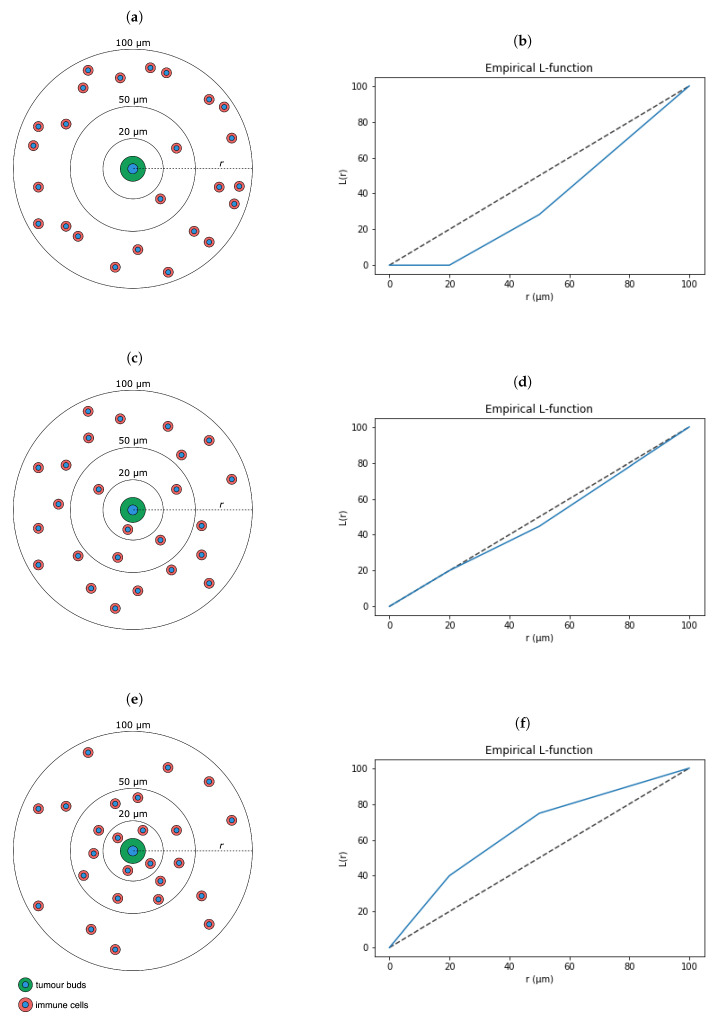
Schematic representation of different immune cell distributions from the nuclear centre of a tumour bud (**a**,**c**,**e**), and their corresponding L function values at different radii (**b**,**d**,**f**). The immune cell population is either (**a**,**b**) dispersed, (**c**,**d**) randomly distributed, or (**e**,**f**) clustered around the tumour bud.

The point coordinates of cell nuclei and immune checkpoint ligand PD-L1 expression were localized across the whole slide images (WSIs), as shown in Figure 2. The Ripley’s K function [44] was adopted to investigate how TBs, PD-L1, and the different populations of immune cells are distributed around each other. In particular, given two populations *X* and *Y*, Ripley’s K function estimates the density of *Y* within a circle of radius *r* around points *X*. As illustrated in Figure 3, assuming a Poisson distribution, the Ripley’s K function can identify whether a population *Y* is dispersed, randomly distributed, or clustered around another population *X*. The *K* function is given as

(1) $$\begin{array}{c} K_{xy}\left(r\right)=\frac{1}{\lambda_{y}}E\left[\mathrm{number}\;\mathrm{of}\;\mathrm{points}\;y\;\mathrm{within}\;a\;\mathrm{distance}\;r\;\mathrm{around}\;a\;\mathrm{point}\;x\right] \end{array}$$

where E[·] encloses all of the points of type *y* within a distance *r* of a randomly selected point of type *x* and $\lambda_{y}$ is the number of points *y* per unit area in the region of interest.

Theoretically, if the point pattern of points *Y* around *X* follows complete spatial randomness, also known as a homogeneous Poisson process, the value of K function is $\pi r^{2}$. The *L* function [45] is a modification of Equation (1), so that the expected output value is *r*, i.e.,

(2) $$\begin{array}{c} L_{xy}\left(r\right)=\sqrt{\frac{K_{xy}\left(r\right)}{\pi }} \end{array}$$

This enables a more intuitive interpretation of the function’s output value in relation to *r*. The *L* function was calculated for TAMs, TILs, and PD-L1 surrounding TBs, as well as the PD-L1 surrounding TAMs and TILs, for a series of increasing distances *r*, where r∈{20,50,100,150,200,250} μm. While some approaches calculate the area under the curve of the *L* function against different *r* values [46], we provided the pairwise spatial distributions between PD-L1, TBs, and the immune populations, directly to the ML classifiers as distinct features.

### 2.7. Binary Survival Analysis

Survival analysis is broadly defined as the analysis of data that involve the time to the occurrence of an event of interest [47]. Herein, the event of interest was the death of an individual due to MIBC. A characteristic of survival analysis is censoring. In our cohort, some patients were right-censored, either because the end of study was reached and the event of interest did not occur, or because the patients succumbed to a cause other than MIBC (abbreviated as OTD-censoring) [47].

Patient survivability was binarized based on a specific time cut-off. Similar to previous works, a 5 year prognosis was investigated [36,48,49]. Patients that succumbed to MIBC within 5 years were denoted as patients with a bad prognosis, whereas those that survived the 5-year cut-off were denoted as patients with a good prognosis. Inevitably, patients that died due to an unrelated cause prior to the prognostic cut-off, i.e., they were part of the OTD-censored data, had to be excluded (19% patient exclusion). It is worth mentioning that removing these patients does not introduce bias, since time to censoring was random, i.e., OTD-censoring was not known *a priori*. A consequence of this approach is that survival analysis was turned into a binary classification problem. Furthermore, due to the removal of censoring, traditional ML models were readily employable.

### 2.8. Model Selection, Algorithm Selection, and Performance Evaluation

Both model selection and algorithm selection attempt to collectively maximize the predictive performance of the final ML model. However, ML algorithms are prone to overfitting, i.e., in finding and using patterns which arise from noise in the data. Such noisy patterns do not generally extend beyond the specific dataset, since noise is typically random. With both a small dataset and a complicated model, the likelihood of overfitting increases. Testing the performance of a trained ML model on unseen data constitutes the mainstay of evaluating the generalizability of an ML model and, therefore, in identifying whether a model has overfitted. As such, a subset of the initial cohort was kept aside as the testing dataset. In particular, using stratified random sampling, two subsets were created: the training set with 75% of the initial data (58 patients), and the testing set with 25% (20 patients). The testing set was only used at the performance evaluation stage to avoid introducing bias to the generalization performance estimate.

Traditionally, model selection is the process by which a ML algorithm is configured. Most ML algorithms come with a number of configuration variables, commonly referred to as hyperparameters. Even though common hyperparameter configurations can be employed, it has been observed that hyperparameter tuning for a specific task can be the key between chance and state-of-the-art models [50]. Since manual tuning can be time-consuming and counter-intuitive in high-dimensional spaces, most ML methodologies adopt automated hyperparameter tuning.

One of the most popular approaches in model selection constitutes a grid search, where each hyperparameter is given a predefined list of values, and the best hyperparameter configuration is selected after evaluating all the combinations. For example, given hyperparameters *A* and *B*, and lists $V_{A}=\left[1,10,100\right]$ and $V_{B}=\left[0.1,0.5\right]$, the following combinations were evaluated under grid search: ∀(A,B)∈ [(1,0.1), (1,0.5), (10,0.1), (10,0.5), (100,0.1), (100,0.5)]. However, as shown by Bergstra and Bengio [51], random sampling provides a better tuning strategy.

In random search, a number of hyperparameter configurations are evaluated by sampling from predefined hyperparameter distributions and densities. For example, given hyperparameters *A* and *B* with $D_{A}∼N\left(0,1\right)$ and $V_{B}=\left[0.1,0.5\right]$, where $D_{A}$ is a standard Gaussian distribution, the following five combinations could have been sampled and evaluated under a random search: ∀(A,B)∈ [(−0.12,0.1), (−0.14,0.5), (−0.94,0.5), (0.44,0.1), (−1.3,0.5)]. In our methodology, 200 hyperparameter configurations were randomly sampled and evaluated for each ML algorithm. A table of the distributions and densities used is provided in Supplementary Table S4.

**Figure 4:**
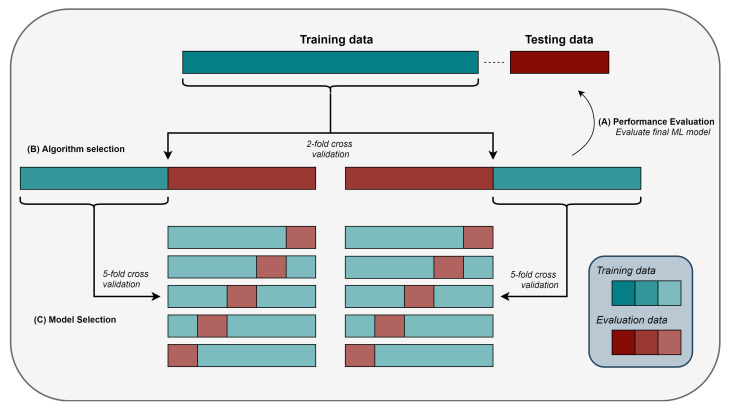
Pictorial representation of nested cross-validation with an independent testing set. (**A**) Performance Evaluation: The best ML algorithm (selected by the outer cross-validation; see (**B**)) was trained on the training dataset and subsequently evaluated on the testing dataset. (**B**) Algorithm Selection: Each ML algorithm (with hyperparameters tuned based on the inner cross-validation; see (**C**)) was trained and tested on the corresponding training and evaluation folds, respectively. The best ML algorithm was selected based on the average performance of both evaluation folds. (**C**) Model Selection: ML models with randomly sampled hyperparameter configurations were trained and tested based on a five-fold cross-validation. The best hyperparameter configuration for each ML algorithm was selected based on the performance on all five evaluation folds.

Not every ML algorithm will perform equally well in different problems and with different data. In addition, there is no theoretical ranking suggesting that one algorithm is better than another [52]. Hence, similar to hyperparameter tuning, algorithm selection is yet another meta-optimization task that needs to be performed for maximizing predictive performance. However, as argued by Bergstra et al. [50]: “Since the performance of a given technique depends on both the fundamental quality of the algorithm and the details of its tuning, it is sometimes difficult to know whether a given technique is genuinely better, or simply better tuned.” Consequently, algorithm selection should involve model selection. Therefore, each ML algorithm was first tuned using five-fold cross-validation and then compared against each other using two-fold cross-validation. This nested cross-validation translates to optimization of the hyperparameters of each ML algorithm twice, and then measurement of their performance on the corresponding evaluation folds (see Figure 4). Subsequently, the ML algorithm which performed better than the others across two different training and validation folds is selected. It is important to highlight how, in most cases, each ML algorithm is evaluated based on two different hyperparameter configurations. Nevertheless, once the ML algorithm was selected, yet another hyperparameter-tuning phase is implemented to find an optimal hyperparameter configuration based on the whole training dataset. Five ML algorithms with different theoretical underpinnings were selected to compete against each other: decision tree (DT), RF, support vector machine (SVM), linear regression (LR), and *k* nearest neighbours (KNN). Finally, a preprocessing step of feature normalisation was added to all classifiers, except DT and RF.

### 2.9. Stratified Sampling

In order to avoid sampling subsets with different class distributions (classes are based on survival with a 5-year cut-off) to the original cohort, stratified sampling was used. Intuitively, when sampling from a dataset with stratification, proportionally, many patients from each class were sampled.—as an example, given a dataset with 75 patients of class $C_{1}$ and 25 patients of class $C_{2}$, a 20% sample would contain 15 patients from class $C_{1}$ and 5 patients from class $C_{2}$.

## 3. Results

### 3.1. Patient Characteristics

A total of 78 patients diagnosed with MIBC were included in this study. The median age of the patients was 68 years (range 29–87 years), with 43 males and 35 females. According to the TNM staging system guidelines [8], our cohort consists of 17 stage II, 29 stage IIIA, 5 stage IIIB, and 27 stage IV patients. Twenty-seven patients had distant metastasis at time of surgery. No positive lymph nodes were found in 57 patients, and 1–2 lymph nodes contained tumour cells in 21 patients. Of the 78 patients, 53 patients died due to bladder cancer. Follow-up information was available for all the patients (range 1–113 months). The clinicopathological characteristics of the cohort are summarised in Table 1.

### 3.2. Fully Automated Feature Extraction

**Figure 5:**
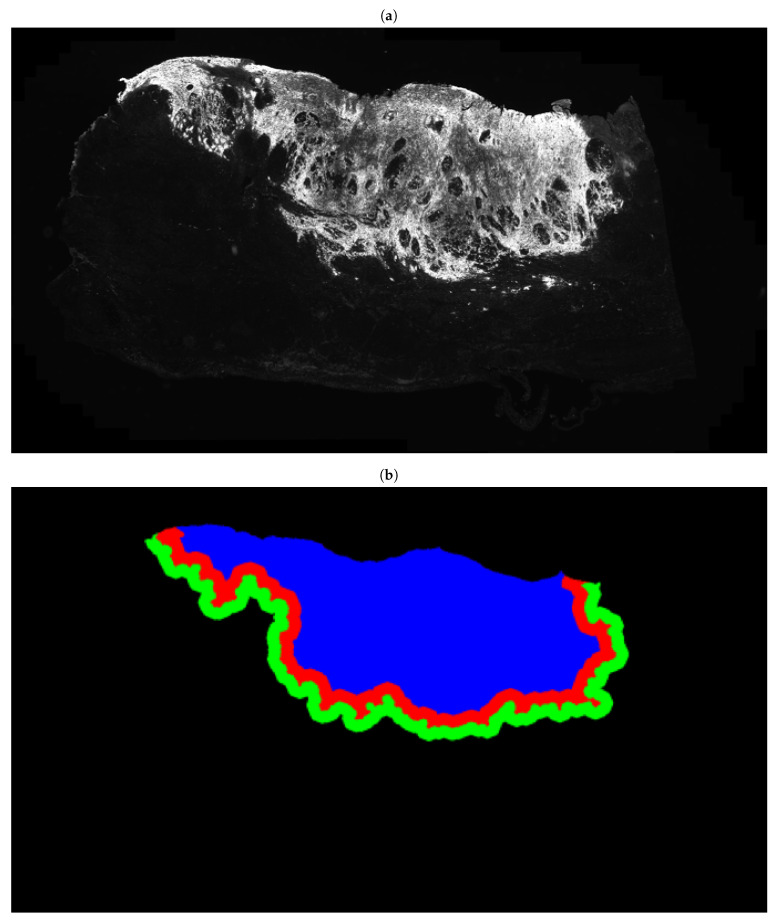
(**a**) Whole-slide immunofluorescence image based on the PanCK channel, (**b**) Segmentation of the corresponding tissue (**a**) into tumour core (blue), invasive frontin (red) and frontout (green) using the PanCK channel.

The entire FFPE tissue section of each MIBC patient was digitized into a WSI, encompassing both muscle-invasive urothelial carcinoma and adjacent benign tissue. Multiplex immunofluorescence, using TSA, enabled the detection of TILs (general CD3 and cytotoxic CD8 T-cells), TAMs (total CD68 macrophages and M2 CD163 macrophages), PD-L1^+^ cells, cell nuclei (Hoechst), and epithelial cancer cells (Pancytokeratin) including TBs across the WSI of each patient. Machine-learning-based image analysis allowed for the localization of each cell, which was subsequently classified depending on its IF signal as either a: (1) TB, (2) M1 macrophage, (3) M2 macrophage, (4) total macrophage, (5) general T cell, (6) cytotoxic T cell, or (7) PD-L1^+^ cell. Based on the above seven classes, a total of 186 quantitative features were extracted from the tumour core and invasive front of each WSI, including the number and density of different cell types, the total size of tumour areas, and the pairwise spatial distributions between immune and cancer cells. The tumour core is defined as the main tumour mass and the invasive front as the border of the tumour core, with a width of 1000 μm (500 μm inside and 500 μm outside of the border, defining the invasive frontin and frontout, respectively), as shown in Figure 5. Feature extraction was performed using Definiens Tissue Phenomics^®^ software (Definiens AG, Munich, Germany) [53,54,55]. A list of all extracted features is provided in Supplementary Table S11.

### 3.3. Feature Space and Feature Selection

In order to capture multiple aspects of the disease, features from both clinical reports and whole-slide immunofluorescence images were quantified. Herein, the number and density of PD-L1-positive and -negative immune cell populations, as well as of TBs from the WSIs are labelled as “image features”. The pairwise spatial distributions between immune cells and TBs are termed “spatial features”. Finally, clinicopathological features such as age, gender, and TNM stage are termed “clinical features”. Information regarding the administration of chemotherapy before or after cystectomy (neoadjuvant or adjuvant) was not provided in a sufficient degree of detail in the authorised release of deidentified clinical records. To maintain the reproducibility of our methodology, chemotherapy was not included as a clinical feature. Altogether, 201 features were quantified—126 image, 60 spatial, and 15 clinical features (the complete feature list is given in Supplementary Table S11). To investigate whether smaller feature spaces result in better ML models, we ran the same ML workflow over different feature sets. In particular, our experiments were based on the following seven feature sets: (i) image, (ii) spatial, (iii) clinical, (iv) image and spatial, (v) image and clinical, (vi) spatial and clinical, (vii) image, spatial, and clinical.

### 3.4. Machine Learning Models and Optimizing Metric

Five ML algorithms with different theoretical underpinnings were selected to investigate whether the extracted features could predict 5-year survivability in MIBC patients; DT, RF, SVM, LR, and KNN. The optimizing metric throughout experimentation was the area under the receiver operating characteristic (AUROC). At the final evaluation phase, classification accuracy, sensitivity, specificity, F1 score, and hazard ratios were also computed for ease of comparative analysis, as shown in Table 2. In order to compute the aforementioned metrics, the optimal threshold values were automatically selected at the final stage based on the training set performance. Hazard ratios and the associated confidence intervals were calculated using univariate Cox regression.

### 3.5. Proposed Ensemble Model

Nested cross validation was implemented to avoid overfitting while maximizing the predictive performance of ML classifiers. In addition, a separate test set was held aside to estimate the generalization performance of the final classifier.

For each of the tested feature sets, the classifier with the highest average AUROC was selected. In case of similar average AUROC between two ML classifiers using the same feature set, we selected the one exhibiting the least variance. The results are shown in Supplementary Table S5. Since multiple classifiers exhibited a similar performance across the different feature sets, instead of employing a single classifier, we combined the best ones into an ensemble model. In particular, our ensemble model consists of a linear support vector machine (LSVM) that uses image features (72.8±0.3 AUROC), a DT that uses image and clinical features (68.8±0.8 AUROC), an LR that uses image and spatial features (70.2±14.7 AUROC), and an RF that uses all features (67.3±5.8 AUROC). Following hyperparameter tuning for each one of the selected classifiers on the whole training set, without cross-validation, our ensemble model was evaluated on the independent testing set, achieving 89.3% AUROC and a highly significant separation of patients into low- and high-risk groups (*p* value $=7\times 10^{-6}$). Patients were classified as high-risk by the ensemble model if two or more of the submodels predicted a bad prognosis.

### 3.6. Pessimistic Bias

The large difference between the generalization estimates of algorithm selection and performance evaluation (see Table S1 and Table 2) can be mostly attributed to pessimistic bias [56]. Given the already small dataset, withholding half of the training dataset for evaluation, due to two-fold cross-validation, increases the chance that an ML model will underfit, i.e., its maximum representation capacity will not be reached [56]. Therefore, the generalization estimate from performance evaluation (Table 2) is more reliable, since the whole training set was used.

### 3.7. Comparing against TNM Staging

**Figure 6:**
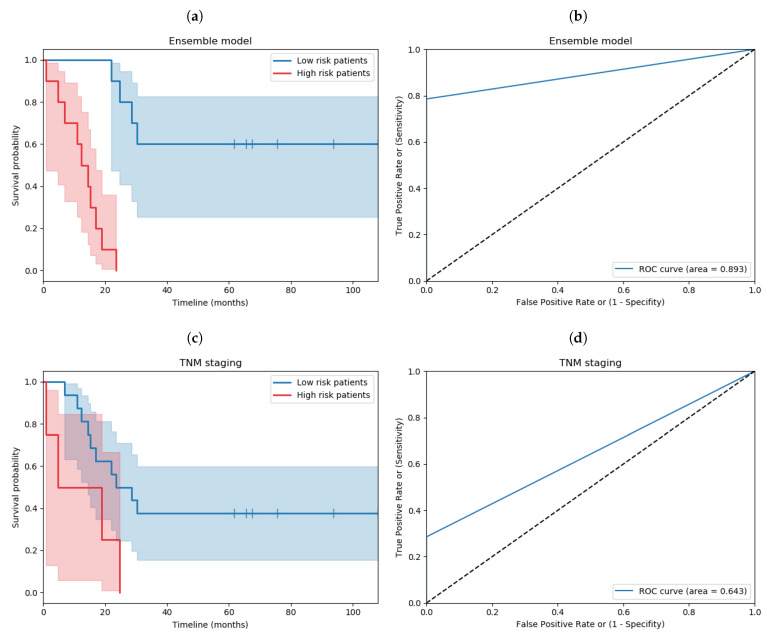
Kaplan–Meier and ROC curves on the testing set for our ensemble model and TNM. Separation was significant based on the (**a**) ensemble model (*p* value $=7\times 10^{-6}$, $N_{LowRisk}=10$ & $N_{HighRisk}=10$) and (**c**) TNM (*p* value =0.04, $N_{LowRisk}=16$ & $N_{HighRisk}=4$). A better AUROC was achieved by (**b**) the ensemble model (AUROC = 0.893) than (**d**) the TNM (AUROC = 0.643).

In order to compare this against the gold standard in clinical practice, TNM, patients had to be stratified into low- and high-risk groups. Based on a pairwise log-rank test comparison in the training dataset (results shown in Supplementary Tables S6–S8), stage II and III patients were considered as the low-risk group, whereas stage IV patients were considered as the high-risk group. The Kaplan–Meier and ROC curves of TNM staging and our ensemble model on the testing set are shown in Figure 6. To allow further comparative analysis, Kaplan–Meier curves of other clinicopathological features, such as age and gender, are shown in Supplementary Figure S3. In addition, the Kaplan–Meier and ROC curves of each submodel of the ensemble model are shown in Supplementary Figure S4.

### 3.8. Post-hoc Analysis of Features

For each classifier of the ensemble model, post-hoc analysis was conducted to reveal the features guiding the survivability prediction. The feature considered at each node of a DT is readily interpretable (see Supplementary Figure S5). For the LR, its coefficients determine the importance, as well as the positive or negative effect, of each feature in patient prognosis. The mean decrease in the Gini index was calculated for each feature of the RF, based on the underlying decision trees [57]. Finally, since the selected SVM had a linear kernel, feature-ranking coefficients were readily available [58]. A threshold was set to filter out features with low feature importance. In particular, the threshold was set to two times the mean importance of all features for the DT, LR, and RF, whereas two times the median importance was used for the LSVM. There were 8, 10, 25, and 16 important features for DT, LR, RF, LSVM, respectively, which are listed in Supplementary Tables S9 and S10. For completeness, Supplementary Tables S12–S14 provide the feature importance values of all features used by LR, RF, and LSVM prior to thresholding. A visualization of the number of intersecting features between the various submodels of our ensemble model is shown in Supplementary Figure S6.

For the LR and LSVM submodels, the high density of TBs in both the invasive frontin and tumour core is highlighted as an indicator of bad prognosis. On the contrary, a high density of CD8^+^, CD3^+^ and CD68^+^ cells is consistently identified as a marker of good prognosis. In addition, a high number of CD3^+^, CD68^+^PD-L1^+^ and CD163^+^PD-L1^+^ cells in the invasive front, as well as the presence of CD3^+^ cells within a distance of 20 μm from TBs, are associated with good prognosis.

For the DT submodel, a low density of CD68^+^, high PD-L1^+^ expression, and high number of TBs (all in frontout) lead to bad prognosis, whereas, given a low density of CD68^+^ and PD-L1^+^ expression in frontout, the prognosis depends on the number of CD68^+^ in frontin. Finally, the majority of the patients with good prognosis had high CD68^+^ in frontout, nonzero PD-L1^+^ expression in core, low CD163^+^ in frontout, and high CD3^+^ in frontout.

Similar to the previous submodels, TBs and CD68^+^ cells were the most important predictors of 5-year prognosis for RF. In addition, RF employed more spatial features than any of the other submodels, including, but not limited to, PD-L1^+^ expression within a distance of 20 μm from TBs and 150 μm from M2 macrophages and the presence of CD3^+^ and CD8^+^ cells within a distance range of 20–50 μm from TB.

## 4. Discussion

In the last decade, advances in the rapidly growing field of tumour immunology have provided further insights into the dynamic nature of the multifaceted immune response throughout the various stages of cancer initiation, evasion, and progression. Concomitantly, multiple research groups have successfully leveraged this new knowledge to improve cancer prognosis, thereby providing evidence for the clinical relevance of immuno-oncology [11]. Accurate patient prognosis is crucial for improving the survival rates of cancer patients, since it is a prerequisite to delivering the most effective treatment for each patient. In fact, multiple papers have shown that the quantitative characterization of the tumour-immune microenvironment components, including TILs, TAMs, and immune checkpoints, can yield information of prognostic relevance [59,60,61]. Particularly, tumour cells surrounded by a large number of prominent intra-tumoural and peri-tumoural TILs and M1 macrophages have been related to better prognosis in several types of cancer [62], whereas a high content of M2 macrophages and TBs has been associated with poorer outcome [60]. In addition, related research has reported the significance of PD-L1 expression on tumour tissues as an independent poor prognostic factor [63]. In this paper, we have investigated, for the first time, the prognostic relevance of immune system biomarkers, TILs, TAMs, TBs, and PD-L1, across whole slide immunofluorescence images of MIBC patients.

H&E is still the most important and commonly used histochemical staining method for studying and diagnosing tissue diseases in histopathology. However, the imaging of H&E stained FFPE tissue has limitations, including the inability to quantify the complex cellular states as well as identify distinct cell populations in the tumour-immune microenvironment. With the advent of whole-slide imaging and the increasing adoption of digital pathology in the clinic [64], multiplex methodologies have the potential to provide significantly more information about the underlying tumour-immune microenvironment than single-marker (i.e., single-label immunohistochemistry) and conventional histochemical-staining-based methodologies [65]. The development of single-protein-based biomarkers to explain patient-level behaviour is hindered by the vast signalling network mediating the heterotypic cell–cell crosstalk between cancer, stromal and immune cells. Instead, with multiplexed methodologies, various proteins can be simultaneously captured on a single tissue sample, encapsulating the tumour-immune architecture from the cellular level down to the subcellular, and ultimately providing more information about the microenvironment.

In our approach, multiplexed immunofluorescence was used to visualize TBs, general and cytotoxic T-cells, M1, M2, and total macrophages, and their co-expression of immune checkpoint ligand PD-L1, in order to quantify their numbers and densities, as well as their pairwise spatial distributions across defined areas (tumour core, invasive frontin and frontout) within a WSI. In the last decade, multiple studies have investigated the topographical distribution of the immune cells within the tumour microenvironment [66,67]. It is known that tumour-infiltrating immune cells are scattered in the tumour core and the invasive front, whereas their density in each tumour region is correlated with patient outcome [68]. Furthermore, the analysis of multiple tumour regions (tumour core and invasive front) was shown to improve the prediction accuracy of patient survival compared to single-region analysis [10,68]. In addition, Immunoscore, a classification system based on the quantification of two lymphocyte populations (CD3 and CD8) within the tumour core and the invasive front of tumour, has been shown to have a prognostic significance superior to that of the TNM staging system in patients with colorectal carcinoma [10,69]. The image, spatial, and clinical features contain a large amount of information about the state of the disease, and collectively portray a more holistic view of each patient’s pathophysiology. We hypothesized that these features can predict the aggressiveness of MIBC, and therefore suggest whether a patient should be considered at low or high risk of disease-specific death.

ML contains a plethora of classifiers that have been employed with success in multiple instances, including diagnosis, segmentation, prognosis, and even therapy planning [35]. In our methodology, survival analysis is turned into a binary classification problem, thus enabling traditional ML algorithms and workflows to be readily employable. In addition, to counter the possibility of overfitting due to having a small dataset and high-dimensional feature space, nested cross-validation with a separate testing set was adopted. The proposed ensemble model significantly surpasses all metrics—AUROC, Accuracy, Specificity, F1 score, Hazard ratio—(89.3%/80.0%/71.4%/83.3%/32.5) the gold standard, TNM staging (64.3%/50.0%/28.6%/44.0%/3.3), as summarized in Table 2. It consists of an LSVM that uses image features, a DT that uses image and clinical features, an LR that uses image and spatial features, and an RF that uses all features.

The results of our study suggest that the characterization of a broad immune cell population, as well as their spatial organization in relation to cancer cells, enables a better estimation of survival compared to the TNM staging system in MIBC patients which, in turn, provides further biological insights. Most of our findings based on whole-slide immunofluorescence images are novel for MIBC, and also corroborate the existing literature on other types of cancer [19,28,31,61,70,71]. In particular, we found that the high content of TBs in the invasive frontin, frontout, and tumour core as well as the low number of CD68^+^ cells and high PD-L1 expression in the invasive frontout, are indicators of bad prognosis [28,30,61,70]. The high density of CD8^+^, CD3^+^, and CD68^+^ cells in the invasive frontin, frontout, and tumour core was associated with good prognosis by our models [19,71]. In addition, the high number of CD3^+^ and CD68^+^ cells, as well as the high number of CD3^+^ cells clustered within a distance of 20 μm from TBs, were linked to good prognosis [31]. Finally, we found that the high density of CD163^+^ cells without PD-L1 expression in frontout is associated with bad prognosis by the DT submodel, whereas the LSVM submodel employed a high number of CD163^+^PD-L1^+^ cells in frontout as an indication of good prognosis.

## 5. Conclusions

In summary, we have demonstrated that ML classifiers using image and spatial features from WSIs, combined with clinical features from medical records, can separate MIBC patients into low- and high-risk groups for 5-year prognosis. The present approach outperforms the current clinical staging system, TNM, reinforcing the importance of the standardized quantification of immunological features across WSIs, as well as the adoption of ML in the clinic. Moreover, our findings show that investigating features from the tumour-immune microenviroment in relation to survival can provide further insights into histopathological studies, thereby contributing to better ways of predicting survivability and enabling a better quality of care.

## Figures and Tables

**Table 1:** Patient cohort characteristics.

Characteristics	Summary
**MIBC patients**	*N* = 78
**Median survival** (range)	19 (1–113) months
**Age**	66±11 years
**Gender**	55% Male; 45% Female
**TNM stage**	
II	17 (22%)
IIIA	29 (37%)
IIIB	5 (6%)
IV	27 (35%)
**Tumour (T)**	
T2	18 (23%)
T3	39 (50%)
T4	21 (27%)
**Node (N)**	
N0	57 (73%)
N1	13 (17%)
N2	8 (10%)
**Metastasis (M)**	
M0	51 (65%)
M1	27 (35%)

**Table 2:** Comparison between our ensemble model and TNM staging. The better performance between the ensemble model and TNM staging for each evaluation metric is shown in bold. * 95% Confidence Interval.

	Training Set		Testing Set	
Evaluation Metrics	Ensemble Model	TNM Staging	Ensemble Model	TNM Staging
AUROC	**98.3**	71.6	89.3	64.3
Accuracy	94.8	65.5	80.0	50.0
Sensitivity	89.5	89.5	100.0	100.0
Specificity	97.4	53.8	71.4	28.6
F1 score	83.3	67.7	83.3	44.0
Hazard ratio	45.9	4.4	32.5	3.3
	(6.2, 341.1) *	(2.3, 8.6) *	(3.9, 270.3) *	(1.0, 11.0) *

## Data Availability

The data and the code of this study are available from the corresponding authors upon request.

## Supplementary Materials

The following are available online at https://www.mdpi.com/article/10.3390/cancers13071624/s1, Figure S1: Tyramide signal amplification spectra for antibody visualisation, Figure S2: Nuclei detection and epithelium segmentation, Figures S3–S4: Kaplan–Meier curves for MIBC prognosis, Figure S5: Diagram of the DT model, Figure S6: Intersecting features between the submodels of the ensemble model, Tables S1–S3: Immunofluorescence primary antibodies and visualization reagents, Table S4: The search space of hyperparameter tuning, Table S5: Nested cross-validation results on the training data set, Tables S6–S8: Pairwise comparison between TNM staging groups, Table S9–S10: Most important features for MIBC prognosis per classifier. Table S11: The complete list of all image, spatial and clinical features, Table S12: The coefficients of logistic regression for all image and spatial features, Table S13: The mean decrease in Gini index for each feature based on the random forest, Table S14: The feature ranking coefficients of linear support vector machine for all image features.
