# Supplementary material for: Assessment of Immunological Features in Muscle-Invasive Bladder Cancer Prognosis Using Ensemble Learning
Source: Cancers (Basel). 2021 Apr 1;13(7):1624. doi: 10.3390/cancers13071624 (PMC8036815; doi:10.3390/cancers13071624)
Supplement: Supplementary file 1 [file cancers-13-01624-s001.zip › cancers-1086945-Supplementary Materials/cancers-1086945-Supplementary Materials.pdf]

Assessment of immunological features in muscle-invasive bladder cancer prognosis

Christos G Gavriel[1,*], Neofytos Dimitriou[2,*], Nicolas Brieu[3], Ines P Nearchou[1], Ognjen Arandjelović[2], Günter Schmidt[3], David J Harrison[1,4], and Peter D Caie[1]

[1]School of Medicine, University of St Andrews, St Andrews, United Kingdom
[2]School of Computer Science, University of St Andrews, St Andrews, United Kingdom
[3]Definiens GmbH, Munich, Germany
[4]NHS Lothian, University Hospitals Division, Edinburgh, United Kingdom
[*]Equal Contribution

Supplementary Information

Table S1. Immunofluorescence primary antibodies.

Antibody	Supplier	Catalogue number	Species	Dilution
CD3	Agilent Technologies	A0452	Rabbit-Polyclonal	1:400
CD8	Agilent Technologies	M7103-Clone C8/144B	Mouse-Polyclonal	1:200
CD68	Cell Signaling	D4B9C	Rabbit-Monoclonal	1:3000
CD163	Cell Marque	MRQ-26	Mouse-Monoclonal	1:3000
PD-L1	Cell Signaling	13684S	Rabbit-Monoclonal	1:100
PanCK	Agilent Technologies	Z0622	Rabbit-Polyclonal	1:100

Table S2. Detection and visualization reagents for target proteins.

Dye	Supplier	Catalogue number	Dilution
FITC	Perkin Elmer	NEL741B001KT	1:100
CY3	Perkin Elmer	NEL744B001KT	1:100
CY5	Perkin Elmer	NEL745B001KT	1:100
Alexa Fluor 750	ThermoFisher Scientific	S21384	1:50

Table S3. Summary of each immunofluorescence target. Visualization and imaging acquisition information.

Target	Dye name	Excitation wavelength (nm)	Emission wavelength (nm)	Exposure time (ms)
Nucleic acid (DNA)	Hoechst	353	465	10
CD3 or CD68	FITC	495	519	10
PD-L1	CY3	548	561	10
CD8 or CD163	CY5	650	673	10
PanCK	CY7	747	773	600

Table S4. The search space of each classifier based on predefined distributions over its hyperparameters.

Classifier	Hyperparameter	Distribution	Values
LSVM	C	Log-uniform	$[\ln(1e-5), \ln(1e2)]$
	Class weight	Categorical	[Balanced, None]
RSVM	C	Log-uniform	$[\ln(1e-5), \ln(1e2)]$
	Gamma	Log-uniform	$1/\text{max\_features} * [\ln(1e-3), \ln(1e3)]$
	Class weight	Categorical	[Balanced, None]
LR	Type of penalty	Categorical	[$l1$, $l2$, Elastic net, None]
	C	Log-uniform	$[\ln(1e-5), \ln(1e2)]$
	L1 ratio	Uniform	[0, 1]
	Class weight	Categorical	[Balanced, None]
DT	Criterion	Categorical	[Gini, Entropy]
	Maximum features	Uniform integer	[1, max_features]
	Maximum depth	Categorical	$[1, 15]$ or None
	Class weight	Categorical	[Balanced, None]
RF	Number of trees	Log-uniform integer	[10, 1000]
	Criterion	Categorical	[Gini, Entropy]
	Maximum features	Uniform integer	[1, max_features]
	Maximum depth	Categorical	$[2, 3, 4$, None]
	Bootstrap	Categorical	[True, False]
	Class weight	Categorical	[Balanced, None]
KNN	K	Log-uniform integer	[1, 20]
	Metric	Categorical	[Balanced, None]
	P	Uniform integer	[1, 6]

Table S5. Results for algorithm selection from the nested cross validation on the training set with AUROC as the performance metric. For each feature space, the best ML classifier is indicated in bold. Amongst the best classifiers of each feature space (in bold), our ensemble model uses those with a marked difference in performance (**).

**Feature Space**	**LR**	**KNN**	**LSVM**	**RSVM**	**DT**	**RF**
**Image**	69.8 ± 13.3	61.8 ± 5.7	$**\mathbf{72.8 \pm 0.3}$	60.8 ± 8.2	63.3 ± 3.3	57.8 ± 3.2
**Clinical**	$\mathbf{58.1 \pm 0.1}$	59.8 ± 7.2	55.9 ± 5.9	48.6 ± 5.6	50.1 ± 2.4	56.6 ± 9.1
**Spatial**	46.8 ± 3.3	46.2 ± 4.7	42.7 ± 1.8	38.0 ± 0.0	$\mathbf{49.2 \pm 3.3}$	43.7 ± 13.0
**Image & clinical**	62.0 ± 0.6	55.3 ± 4.4	56.5 ± 8.5	50.1 ± 11.5	$**\mathbf{68.8 \pm 0.8}$	70.4 ± 7.9
**Image & spatial**	$**\mathbf{70.2 \pm 14.7}$	59.9 ± 2.4	49.0 ± 2.5	58.6 ± 11.9	51.0 ± 1.5	64.1 ± 7.6
**Clinical & spatial**	44.3 ± 9.8	49.4 ± 3.2	$\mathbf{57.9 \pm 2.4}$	47.1 ± 4.4	44.8 ± 2.7	46.6 ± 15.4
**Image & clinical & spatial**	56.3 ± 0.2	55.0 ± 4.0	60.2 ± 8.2	61.4 ± 4.6	66.6 ± 9.1	$**\mathbf{67.3 \pm 5.8}$

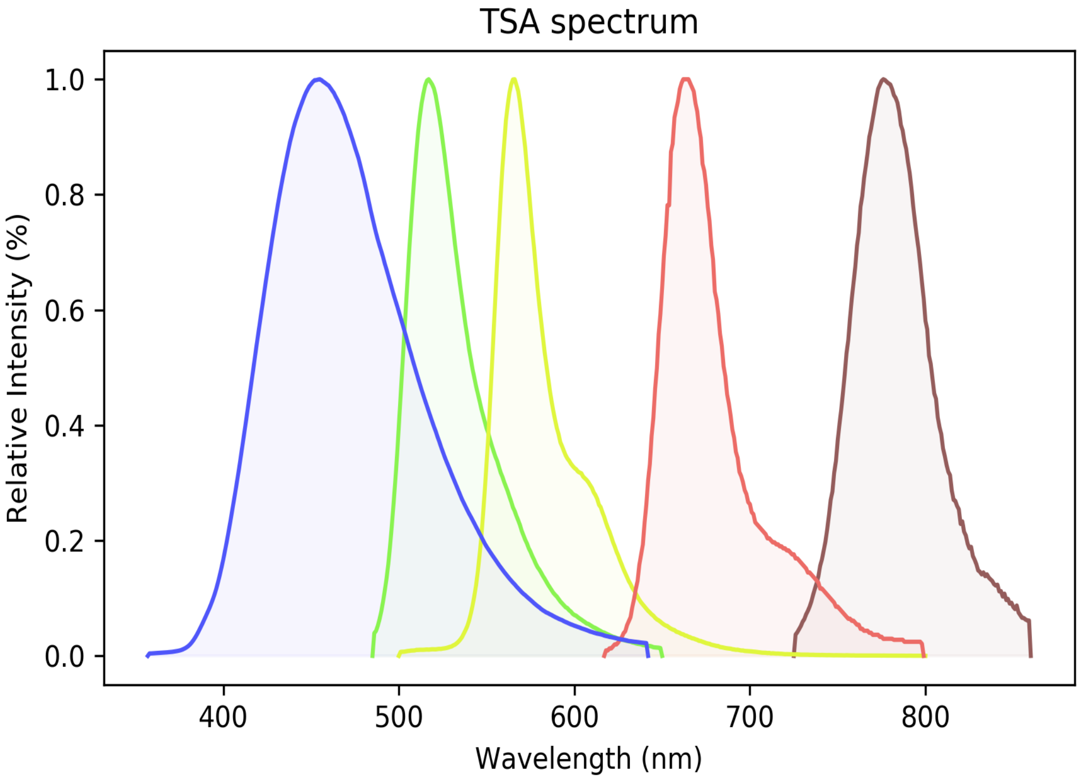

Figure S1. Tyramide signal amplification spectra for antibody visualisation. *Hoechst: Blue, FITC: Green, CY3: Yellow, CY5: Soft red, Alexa Fluor 750 (CY7): Dark red .*

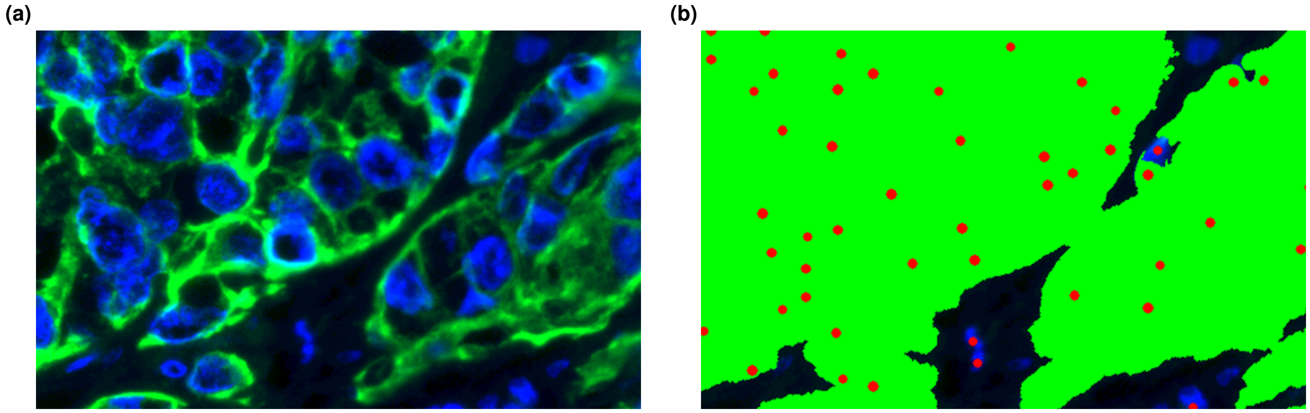

Figure S2. (a) Immunofluorescence region visualised using the PanCK (green) and Hoechst (blue) channels, (b) The corresponding epithelium segmentation mask of (a) along with the detected cell nuclei in red dots.

Table S6. Pairwise comparison between TNM staging groups. Stages IIIA and IIIB were merged due to having the largest p value.

TNM	II	IIIA	IIIB
IIIA	0.33792	-	-
IIIB	0.50382	0.93012	-
IV	0.00087	0.00317	0.07361

Table S7. Pairwise comparison between TNM staging groups. Stages II and IIIA-IIIB were merged due to having the largest *p* value.

TNM	II	IIIA-IIIB
IIIA-IIIB	0.21458	-
IV	0.00034	0.00034

Table S8. Pairwise comparisons using log-rank test between TNM staging groups. Since the *p* value was significant, the two stages were not merged.

TNM	II-IIIA-IIIB
IV	1.6e-06

Table S9. The features that contribute to a good and bad prognosis according to the LR and the LSVM. L(x,y,r): the L function value of *y* in respect to *x* for distance *r*.

**Classifiers**	**Bad Prognosis**	**Good Prognosis**
**LR**	Density of TB frontin/core	Density of $CD8^+$ frontin/frontout/core<br>Density of $CD3^+$ frontout/core<br>Density of $CD68^+$ frontin/frontout<br>L(TB, $CD3^+$, 20)
**LSVM**	Density of TB frontin/core	Density of $CD8^+$ frontin/frontout/core<br>Density of $CD3^+$ frontin/frontout/core<br>Density of $CD68^+$ frontin/frontout/core<br>Number of $CD3^+$ frontin<br>Number of $PDL1^+CD163^-CD68^+$ frontout<br>Number of $PDL1^+CD163^+CD68^+$ frontout

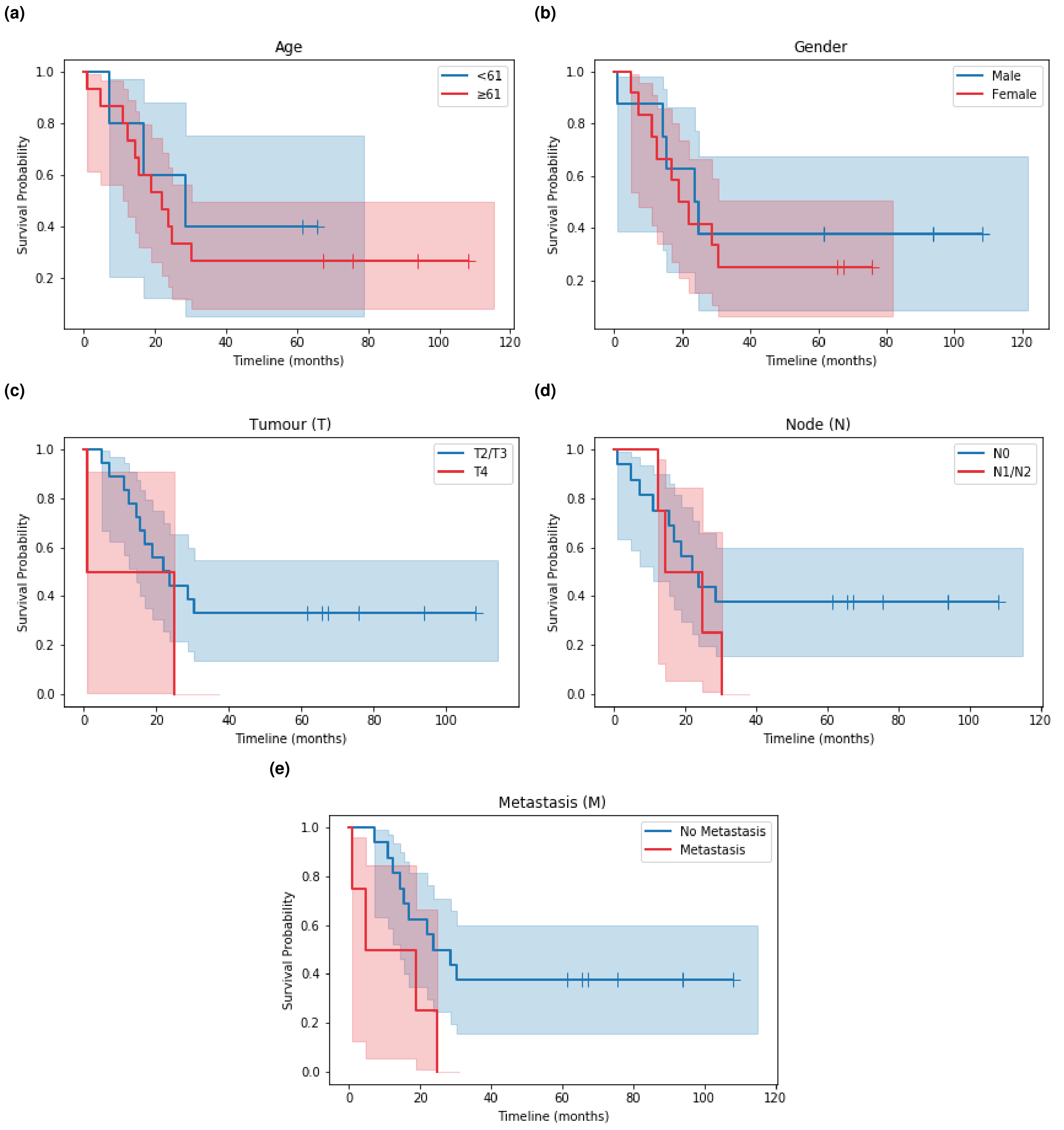

Figure S3. Kaplan-Meier curves of (a) Age (p value $= 0.57$, $N_{<61} = 5$ & $N_{\geq 61} = 15$), (b) Gender (p value $= 0.62$, $N_{Female} = 12$ & $N_{Male} = 8$), (c) Tumour (p value $= 0.25$, $N_{T2/T3} = 10$ & $N_{T4} = 2$), (d) Node (p value $= 0.36$, $N_{N0} = 16$ & $N_{N1/N2} = 4$), and (e) Metastasis (p value $= 0.04$, $N_{No} = 16$ & $N_{Yes} = 4$).

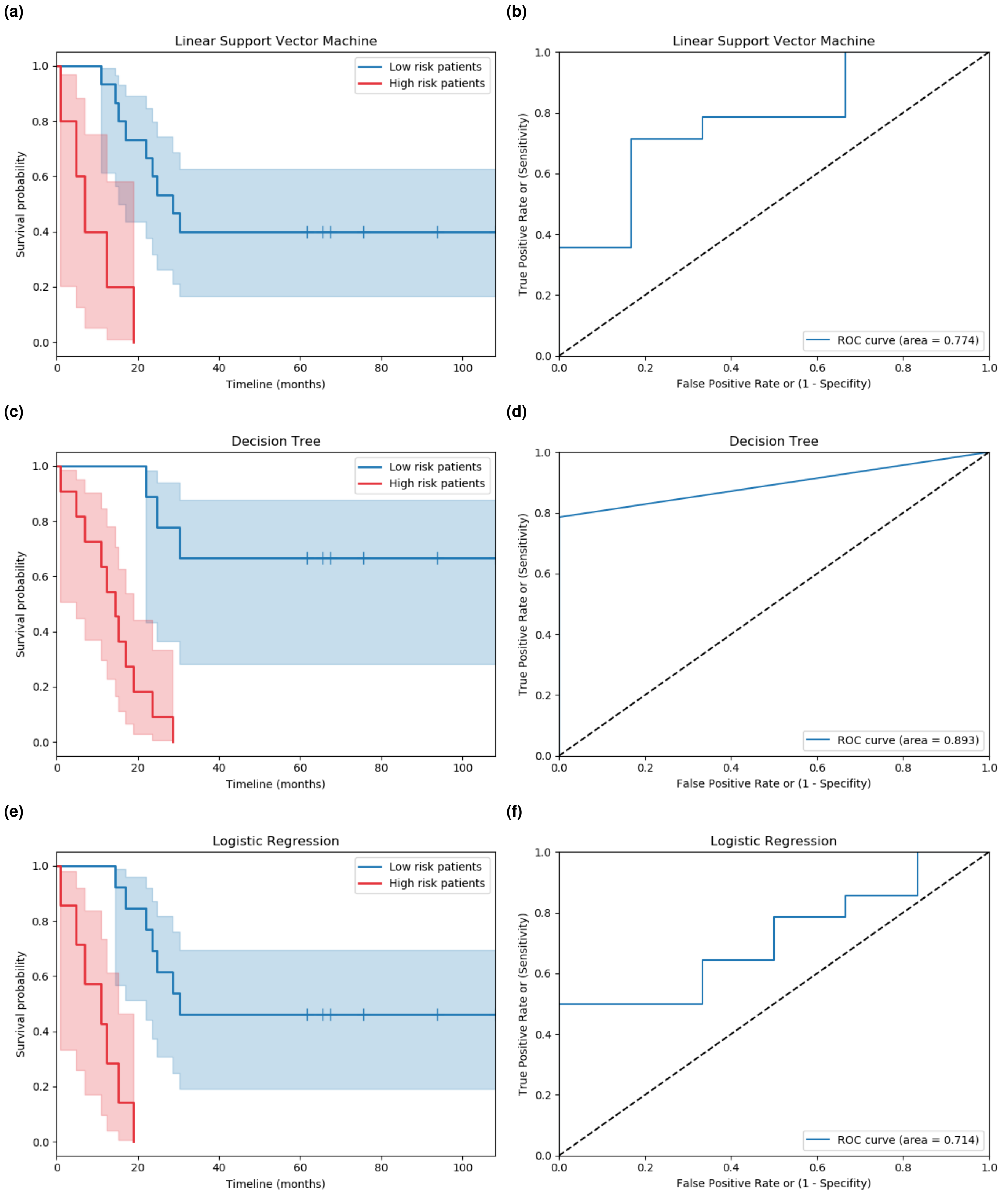

(a)
Linear Support Vector Machine
Low risk patients
High risk patients
Survival probability
Timeline (months)
(b)
Linear Support Vector Machine
ROC curve (area = 0.774)
True Positive Rate or (Sensitivity)
False Positive Rate or (1 - Specifity)
(c)
Decision Tree
Low risk patients
High risk patients
Survival probability
Timeline (months)
(d)
Decision Tree
ROC curve (area = 0.893)
True Positive Rate or (Sensitivity)
False Positive Rate or (1 - Specifity)
(e)
Logistic Regression
Low risk patients
High risk patients
Survival probability
Timeline (months)
(f)
Logistic Regression
ROC curve (area = 0.714)
True Positive Rate or (Sensitivity)
False Positive Rate or (1 - Specifity)

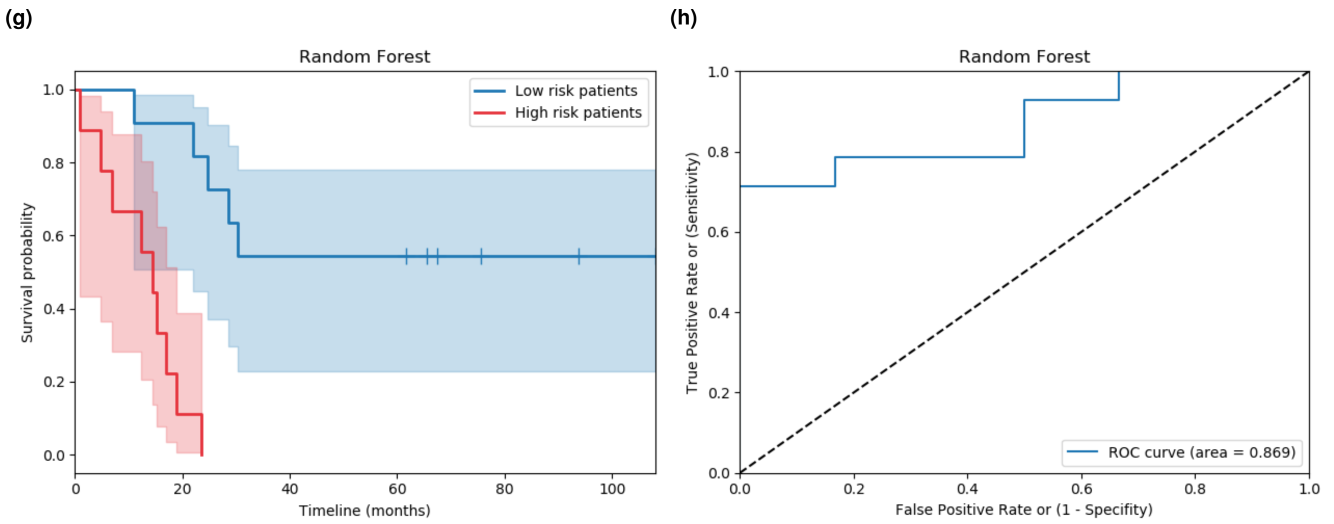

Figure S4. Kaplan-Meier and ROC curves on the testing set for each of the ML classifiers used in the ensemble model. (a–b) Image features (p value $= 6e-05$, $N_{LowRisk} = 15$ & $N_{HighRisk} = 5$), (c–d) image and clinical features (p value $= 1e-04$, $N_{LowRisk} = 9$ & $N_{HighRisk} = 11$), (e–f) image and spatial features (p value $= 5e-05$, $N_{LowRisk} = 13$ & $N_{HighRisk} = 7$), (g–h) image, clinical, and spatial features (p value $= 8e-06$, $N_{LowRisk} = 11$ & $N_{HighRisk} = 9$).

Table S10. The most important features for estimating patient prognosis by the DT and the RF. L(x,y,r): the L function value of y in respect to x for distance r.

Classifiers	Important Features
**DT**	Number of PD-L1^{+} frontout
	Density of CD163^{+} frontout
	Density of PD-L1^{+}CK^{+} core
	Number of TB frontout
	Density of CD3^{+} frontout
	Number of CD68^{+} frontout
	Density of CD68^{+} frontin/frontout
**RF**	Number of CD68^{+} frontout
	Density of CD68^{+} frontin/frontout/core
	Number of CD3^{+} core
	Density of CD3^{+} frontout
	Number of CD8^{+} frontout/core
	Density of CD8^{+} frontout/core
	Number of TB core
	Density of PD-L1^{+} frontout
	Density of PD-L1^{+}CK^{+} core
	Density of PD-L1^{+}CK^{-} frontin/frontout
	Number of CD163^{+}CD68^{+} frontout
	Number of NucleiCK^{+} frontin
	TNM IIIA
	TNM IV
	L(TB, CD3^{+}, 20)
	L(TB, CD8^{+}, 20)
	L(TB, PD-L1^{+}, 20)
	L(TB, CD8^{+}, 50)
	L(CD163^{+}, PD-L1^{+}, 150)

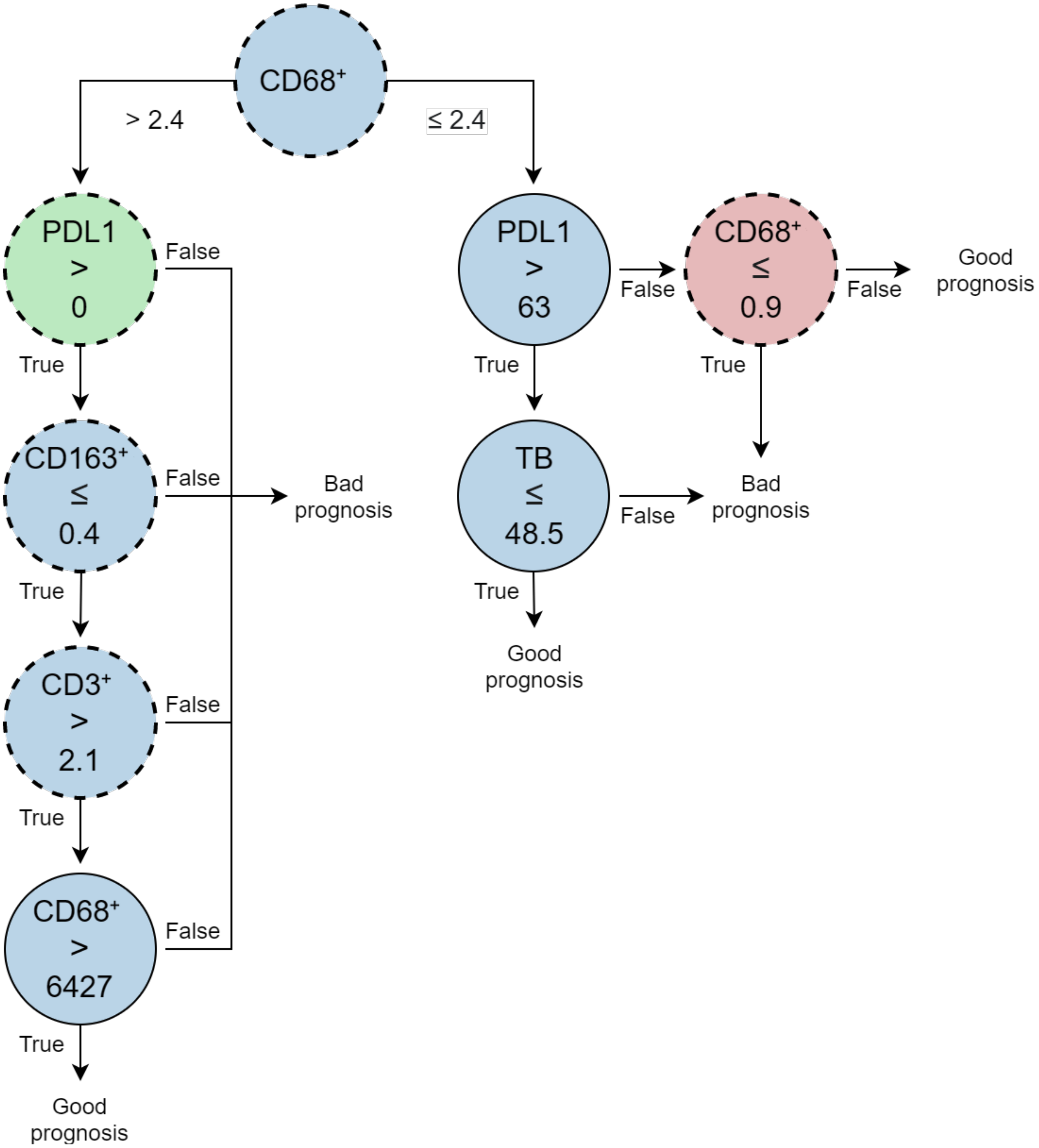

Figure S5. Diagram of the DT model employed by the ensemble model. *Solid-line circles: number of cells, dashed-line circles: density of cells. Tumour core in green, frontin in red and frontout in blue.*

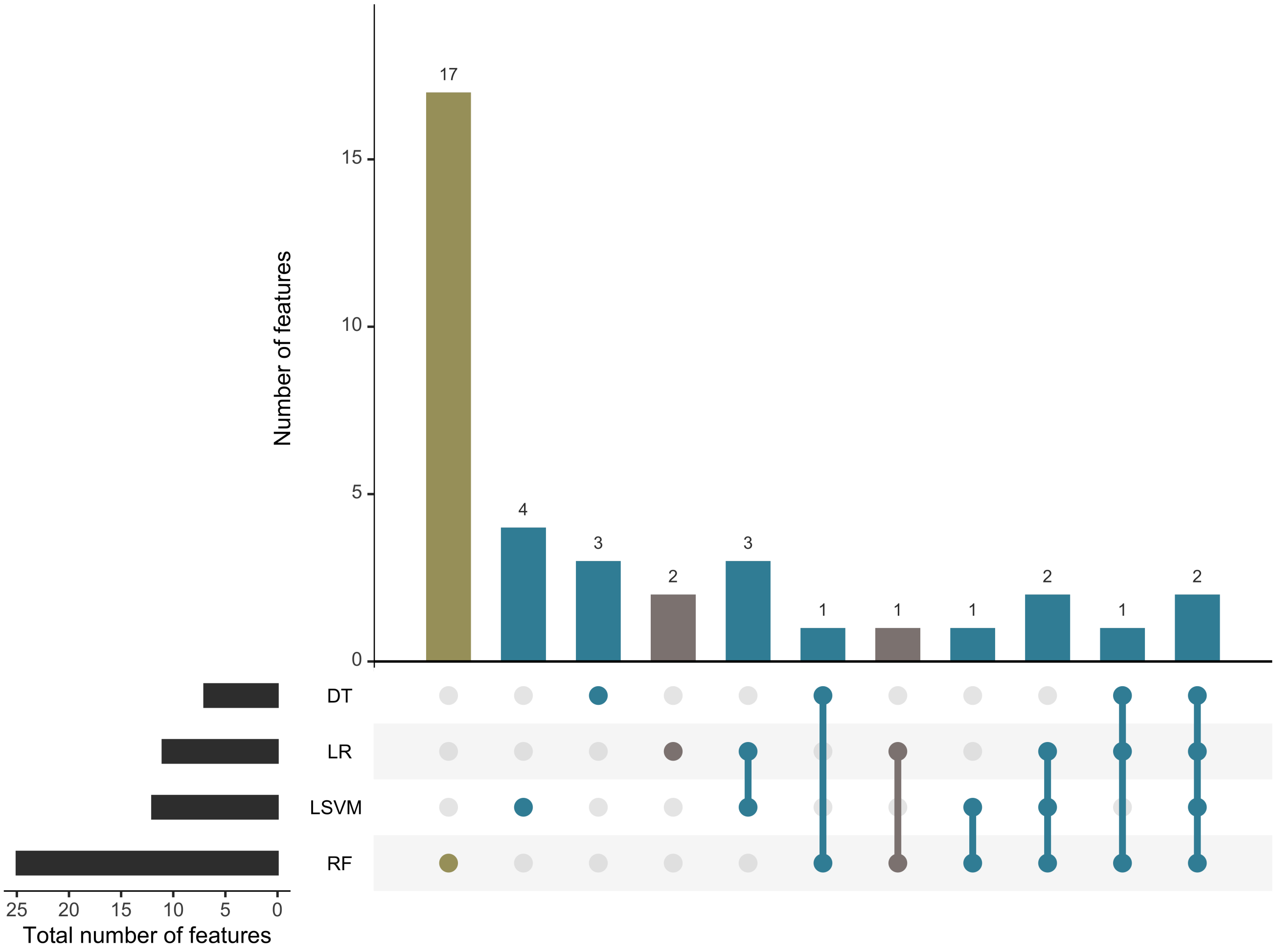

Figure S6. Intersecting features between the various submodels of our ensemble model. Gray denotes image and spatial features, blue denotes image features, and mustard denotes image, clinical, and spatial features. The x-axis is a matrix layout that shows the feature intersections between the submodels.
